# Programmed cell death ligand 1(PD-L1) association in metastatic and non-metastatic oral squamous cell carcinoma: clinicopathologic and immunohistochemical study

**DOI:** 10.1038/s41598-026-60283-6

**Published:** 2026-07-04

**Authors:** Eman M. Kamel, Doaa A. M. Esmaeil, Ramy A. Abdelsalam, Azza A. El-Sisi

**Affiliations:** 1 Assistant Lecturer of Oral Pathology, Faculty of Dentistry, Mansoura University, Mansoura, Egypt; 2 Associate Professor of Oral Pathology,Faculty of Dentistry, Mansoura University, Mansoura, Egypt; 3 Lecturer of Pathology, Faculty of Medicine, Mansoura University, Mansoura, Egypt; 4 Professor of Oral Pathology, Faculty of Dentistry, Mansoura University, Mansoura, Egypt

**Keywords:** OSCC, Immunohistochemistry, PD-L1, Metastatic OSCC, Cancer, Oncology

## Abstract

Oral Squamous Cell Carcinoma (OSCC) is considered a highly immunosuppressive malignancy largely mediated by the Programmed Cell Death 1/Programmed Cell Death Ligand 1(PD-1/PD-L1) axis. The interaction between PD-L1 expressed on tumor cells and PD-1 receptors on T-cells results in T-cell dysfunction, exhaustion, and immune evasion within the tumor microenvironment. This study aimed to evaluate PD-L1 expression in primary non-metastatic OSCC, primary metastatic OSCC, and nodal metastatic OSCC, as well as to investigate its association with different available clinicopathological parameters. Immunohistochemical staining was performed to retrospectively evaluate PD-L1 expression in 30 archival paraffin-embedded OSCC specimens retrieved from the Department of Oral Pathology, Faculty of Dentistry, and the Oncology Center, Faculty of Medicine, Mansoura University. PD-L1 immunoreactivity was evaluated using a semi-quantitative scoring system based on both the staining intensity and the percentage of positively stained cells. The percentage of immunopositive cells was scored as stated: 0 (0%); 1 (< 25%); 2 (25–50%); 3 (50–75%); and 4 (> 75%). Staining intensity was graded as follows: (0 = negative); (1 = weak); (2 = moderate); and (3 = strong). A combined immunoreactivity score was calculated by adding the percentage and the intensity for each case (range 0–7). The final score was categorized as follows: 0 (negative); 1–3 (weak); 4–7 (strong). Statistical analysis was conducted to determine significant differences and correlations between PD-L1 expression and clinicopathological parameters using the Chi-square test, Monte Carlo test, one-way ANOVA, Student’s t-test, and Fisher’s exact test. The p-value < 0.05 was considered statistically significant. PD-L1 immunopositivity was detected in all OSCC cases (100%). A statistically significant difference was observed among the different studied groups (*p* < 0.001), with the strongest PD-L1 expression detected in both primary metastatic and nodal metastatic OSCC. Strong PD-L1 expression showed a significant association with patient age (*p* = 0.024). Additionally, a significant correlation was identified between PD-L1 expression and the depth of tumor invasion (*p* < 0.001). PD-L1 expression may have a potential role in tumor progression of OSCC.

## Introduction

Oral squamous cell carcinoma is considered to be the most common tumor of the oral cavity, accounting for more than 90% of all oral malignancies^1^. Globally, in 2020, there were 377,713 new cases of lip and oral cavity cancer, making the disease the fifth most common carcinoma. 177,757 of the patients with this disease died^2^. It is a multifactorial malignant disease arising from the oral mucosa and carries a poor prognosis that has shown minimal improvement over the past few decades^3^. Additionally, due to poor survival rates, treatment is often associated with high morbidity as the disease affects facial tissues that exhibit notable esthetic and functional loss after treatment^4^. Failure to efficiently treat OSCC has fatal outcomes due to the possible loco-regional recurrence and distant metastasis. Lymph node metastasis (LNM) is a significant predictor of cancer-related outcomes in OSCC^5^.

It has been suggested that oral carcinoma develops via progressive accumulation of key molecular changes in tumor suppressor genes and oncogenes, perhaps in a specific sequence, until the final required alterations complete the necessary cancer genotype and initiate invasion^6^. This is assisted by a gradual increase in mutations and chromosomal disturbances in dysplastic lesions^7^. Among the tumor microenvironment cellular components, the immune cells are a crucial regulator of tumor biology, having the capacity to support or inhibit the development, growth, invasion, and metastasis of tumors^8^. The adaptive immune cells, including T and B lymphocytes, and innate immune cells, like macrophages, neutrophils, mast cells, dendritic cells, and natural killer cells, have all been found in the tumor microenvironment^9^. The T lymphocyte, particularly its ability to mediate antigen-directed cytotoxicity, has become a major focus in cancer immunology^10^.

OSCC has been considered to be a very immunosuppressive cancer, and there is increasing knowledge that treatment failure in OSCC may be related to the dysfunction of the immune system^11^. An immunosuppressive network is instituted through interactions between cancer cells and immune cells in the microenvironment, which results in cancer cells evading immune-mediated attack. In some conditions, the tumor cells can evade the defensive influence of the immune system, thus hindering its effect and continuing growth and progression. This concept is known as cancer immunoediting^12^, which comprises the obtained abilities for maintaining proliferative signaling, escaping growth suppressors, resistance to cell death, facilitating replicative immortality, inducing angiogenesis, stimulating metastasis and invasion, reprogramming metabolism of cells, tumor-promoting inflammation, and immune destruction avoidance: a process termed immune tolerance^13^. This allows the tumor cells to multiply unrestrained by the immune system via inhibitory signaling pathways named immune checkpoints^14^. PD-1 is one of the very important immune checkpoints through the axis of the PD-1 receptor and its ligand, PD-L1^15^. So, tumor cells exploit these inhibitory pathways to evade host immunosurveillance by upregulation of PD-L1^16^.

In normal circumstances, the PD-1/PD-L1 signaling pathway controls both the induction and preservation of peripheral tolerance. It regulates functions of T-cell effector during varied physiological responses, involving acute and chronic infections, autoimmunity, and cancer. After T-cell activation, PD-1/PD-L1 interactions may restrict self-reactive T-cell proliferation and cytokine production^17^. By eluding tumor-neutralizing immune surveillance, PD-1 and its ligand PD-L1 play a critical role in tumor development and surviving in the tumor microenvironment^18^. It has been detected that PD-1 is expressed on a diversity of immune cells, tumor-infiltrating lymphocytes (TIL), and tumor cells. Due to the interaction of PD-L1 with PD-1 on T-cells in the tumor, T-cell malfunction, exhaustion, neutralization, and the generation of interleukin-10 are explained (IL10)^19^. Consequently, tumor overexpression of PD-L1 serves as a mechanism of immune evasion from T-cell-triggered killing. So, tumor cells exhibit more aggressive behavior and secrete numerous pro-inflammatory cytokines due to T-cell exhaustion^20^. Several studies revealed the prognostic value of monitoring the expression of PD-L1 in various tumors. Moreover, the PD-1 and its ligand biological importance proposed that the potential of therapy via manipulation of PD-1 pathway against several human diseases was significant^21–23^. Despite the growing interest in the role of PD-L1 in tumor immune evasion, the pattern of its expression across different stages of OSCC progression, particularly between primary non-metastatic tumors, primary tumors with metastasis, and nodal metastatic lesions, remains insufficiently clarified. Moreover, limited data are available regarding the relationship between PD-L1 expression and clinicopathological parameters in these distinct categories. Metastasis represents one of the most critical determinants of prognosis in OSCC, particularly lymph node metastasis, which significantly affects patient survival and treatment outcomes. Therefore, evaluating PD-L1 expression in both primary and metastatic OSCC lesions may provide further insight into tumor progression and the potential role of immune checkpoint pathways in metastasis.

### Aim of the study

The present study aimed to evaluate the differential immunohistochemical (IHC) expression of PD-L1 in squamous epithelial malignancy in the following lesions: Primary non-metastatic OSCC, Primary metastatic OSCC, and Nodal metastatic OSCC. It also aimed to determine the relation between PD-L1 expression and the available clinical data of the studied groups.

## Materials and methods

### Specimen selection and data retrieval

This study was approved by the Ethics Committee of the Faculty of Dentistry, Mansoura University, Egypt, under the code number: A23080622op. All methods were carried out in accordance with relevant guidelines and regulations. As a retrospective study conducted on 30 formalin-fixed paraffin-embedded (FFPE) tissue specimens, the requirement for informed consent was waived by the Ethics Committee. Sections retrieved from the archival files of the Department of Oral Pathology, Faculty of Dentistry, and the Oncology Center, Faculty of Medicine, Mansoura University. The selected sample of the present work was designed to include OSCC cases classified according to histological grade^24^ to form the following groups: Group A: Primary non-metastatic OSCC (10 paraffin sections). Group B: Primary metastatic OSCC (10 paraffin sections). Group C: Nodal metastatic OSCC (10 paraffin sections).

Cases were selected according to the criteria of their metastatic status based on the available clinical records and histopathological reports. Non-metastatic OSCC cases were defined as primary tumors without evidence of regional lymph node metastasis at the time of diagnosis. Primary metastatic OSCC cases were defined as primary tumors associated with confirmed regional lymph node metastasis. Nodal metastatic OSCC cases were defined as metastatic deposits identified within cervical lymph nodes and histopathologically diagnosed as metastatic squamous cell carcinoma originating from the primary oral lesion.

Cases were excluded if the paraffin blocks showed insufficient tumor tissue, poor preservation, or extensive necrosis that could interfere with immunohistochemical evaluation. Specimens with incomplete clinical or pathological data were also excluded. In addition, recurrent tumors or cases that had received prior chemotherapy or radiotherapy before biopsy were excluded to avoid potential effects on PD-L1 expression.

### Clinical data

Clinical as well as demographic data of all studied cases were retrieved from patients’ medical records regarding age, gender, site, and pain of OSCC cases.

### Histological examination

From each of the selected paraffin blocks, two Sect.  (4 μm thickness) were cut and stained with hematoxylin and eosin stain to review the diagnosis of the studied cases regarding the histologic grade of carcinoma.

### Immunohistochemical staining

Paraffin sections for IHC were mounted on Opti Plus slides that are electrically charged to permit the tissue sections’ adhesion to the slide surfaces. These sections were subjected to IHC staining with the PD-L1, a rabbit monoclonal antibody (clone QR001, Quartett Biotechnologie, Germany). The antibody was supplied as a ready-to-use, prediluted solution.

In light of the manufacturer’s instructions, IHC was carried out. Formalin-fixed paraffin-embedded tissue sections were rehydrated using graded ethanol and distilled water after being deparaffinized in xylene. Endogenous peroxidase activity was blocked using 3% hydrogen peroxide for 10 min. In citrate buffer (pH 6.0), antigen retrieval was carried out by heating near boiling for 10–20 min, followed by cooling for 30 min at room temperature. Phosphate-buffered saline (PBS) was used to rinse the sections.

Slides underwent incubation for 60 min using ready-to-use anti-PD-L1 primary antibody in a humid chamber, followed by incubation with HRP polymer detection system in line with the manufacturer’s protocol. Between steps, PBS was used to wash the sections. Immunoreactivity was detected using a chromogen called diaminobenzidine (DAB). Sections were counterstained with Harris hematoxylin, dehydrated in increasing alcohol grades, cleaned in xylene, and mounted.

### Control slides

#### Positive control slides

According to the manufacturer’s instructions, the tissue sections served as positive control were the paraffin-embedded rat tonsil.

#### Negative control slides

The negative control slides were run with the test where the step of monoclonal antibody was replaced by the addition of non-immune serum.

These negative control tissue sections were important to assess the background staining.

### Evaluation and scoring of immunohistochemical reaction

Immunohistochemically stained sections were examined using a light microscope (Olympus CX31) at ×200 magnification. The evaluation was performed independently by two observers. Inter-observer agreement between the two independent observers was assessed using Cohen’s kappa coefficient. For each section, three representative fields rich in lesional cells were selected. PD-L1 expression was assessed using a semiquantitative scoring system based on two parameters: the percentage of immunopositive cells and staining intensity.

The percentage of positively stained cells was scored as follows:

0 (0% positive cells),

1 (< 25%),

2 (25–50%),

3 (50–75%),

4 (> 75%).

Staining intensity was graded as:

0 (negative),

1 (weak),

2 (moderate),

3 (strong).

A combined immunoreactivity score (IRS) was calculated for each case by adding the percentage score and the intensity score, resulting in a total score ranging from 0 to 7 as follows:

Score 0: negative expression.

Score 1–3: weak expression.

Score 4–7: strong expression.

Cut-off values for positivity of PD-L1 expression were defined as follows:

Cases with a combined score ≥ 1 were considered positive for PD-L1 expression, while a score of 0 was considered negative. Only the final combined scores were used for statistical correlation analysis^25,26^.

### Statistical analysis and data interpretation

The statistical analyses were performed using IBM SPSS Statistics for Windows, Version 25.0 (IBM Corp., Armonk, NY, USA). The software is available at: https://www.ibm.com/products/spss-statistics. Qualitative data were expressed as numbers and percentages, while quantitative data were presented using mean ± standard deviation (SD) for normally distributed data after testing normality using the Shapiro-Wilk test. A p-value ≤ 0.05 was considered statistically significant. To compare the qualitative data among groups as suitable, tests were analyzed such as Chi-Square, Fisher’s exact, and Monte Carlo, while to compare two independent groups for normally distributed data, the Student t-test was used. More than two independent groups were compared using the one-way analysis of variation (ANOVA) test, and pairwise comparisons were found using the Post Hoc Tukey test.

### Sample size calculation

**Fig. 1:**
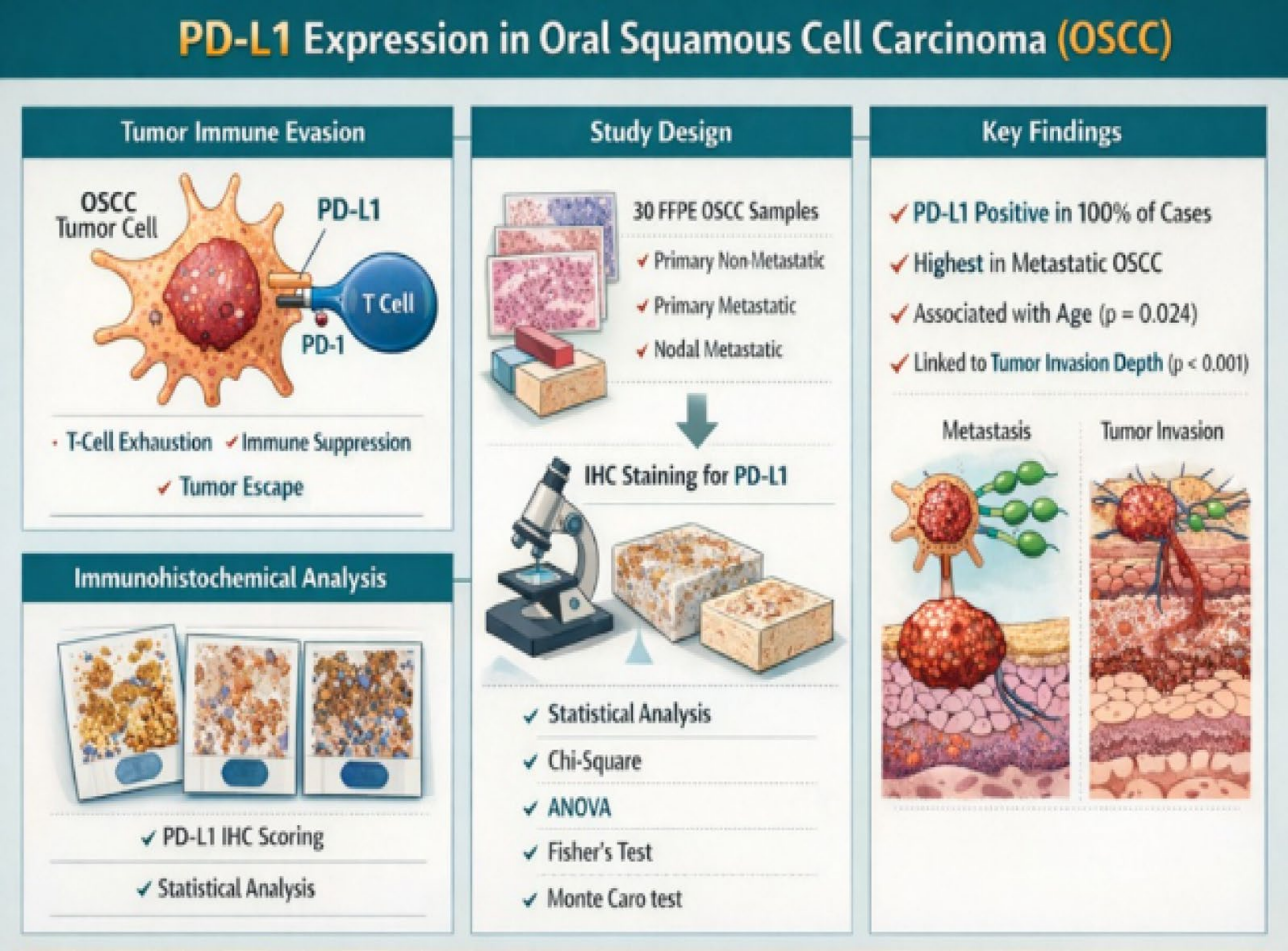
Study design illustration.

Sample size calculation was performed using G*Power: Statistical Power Analyses for Windows, Version 3.1.9.2 (Heinrich Heine University Düsseldorf, Germany; formerly University of Kiel, Germany). The software is available at: https://www.psychologie.hhu.de/en/working-groups-and-labs/general-psychology-and-work-psychology/gpower. The effect size f was 0.69 using an alpha (α) level of 0.05 and a Beta (β) level of 0.20, i.e., power = 80%; the minimum required sample size was estimated to be 30 cases (Fig. 1).

## Results

### Clinicopathological parameters

As shown in Table 1, the study included 30 OSCC cases divided into three groups. The mean ± SD age of patients was 52.40 ± 13.85 years in the non-metastatic group, 58.50 ± 12.35 years in the metastatic group, and 58.20 ± 11.27 years in the nodal metastatic group, with no statistically significant differences among groups. Females were slightly predominant across all groups. The most frequently affected site was the tongue (13/30). Approximately half of the cases presented with pain, while the remaining cases were asymptomatic (Table 1).

**Table 1:** Clinicopathological parameters between different studied groups.

Clinical variables	Group A: Primary non-metastatic *n* = 10 (%)	Group B: Primary metastatic *n* = 10 (%)	Group C: Nodal metastatic *n* = 10 (%)
Age/years			
mean ± SD	52.40 ± 13.85	58.50 ± 12.35	58.20 ± 11.27
Patients’ Sex			
Male: Female	5:05	3:07	3:07
Site			
Cheek	2(20)	2(20)	5(50)
Mandible	2(20)	1(10)	1(10)
Palate	1(10)	2(20)	1(10)
Tongue	5(50)	5(50)	3(30)
Pain			
absent	5(50)	6(60)	5(50)
present	5(50)	4(40)	5(50)

Used test: One-way ANOVA test, Monte Carlo test, SD: standard deviation.

### Histopathological results

**Fig. 2:**
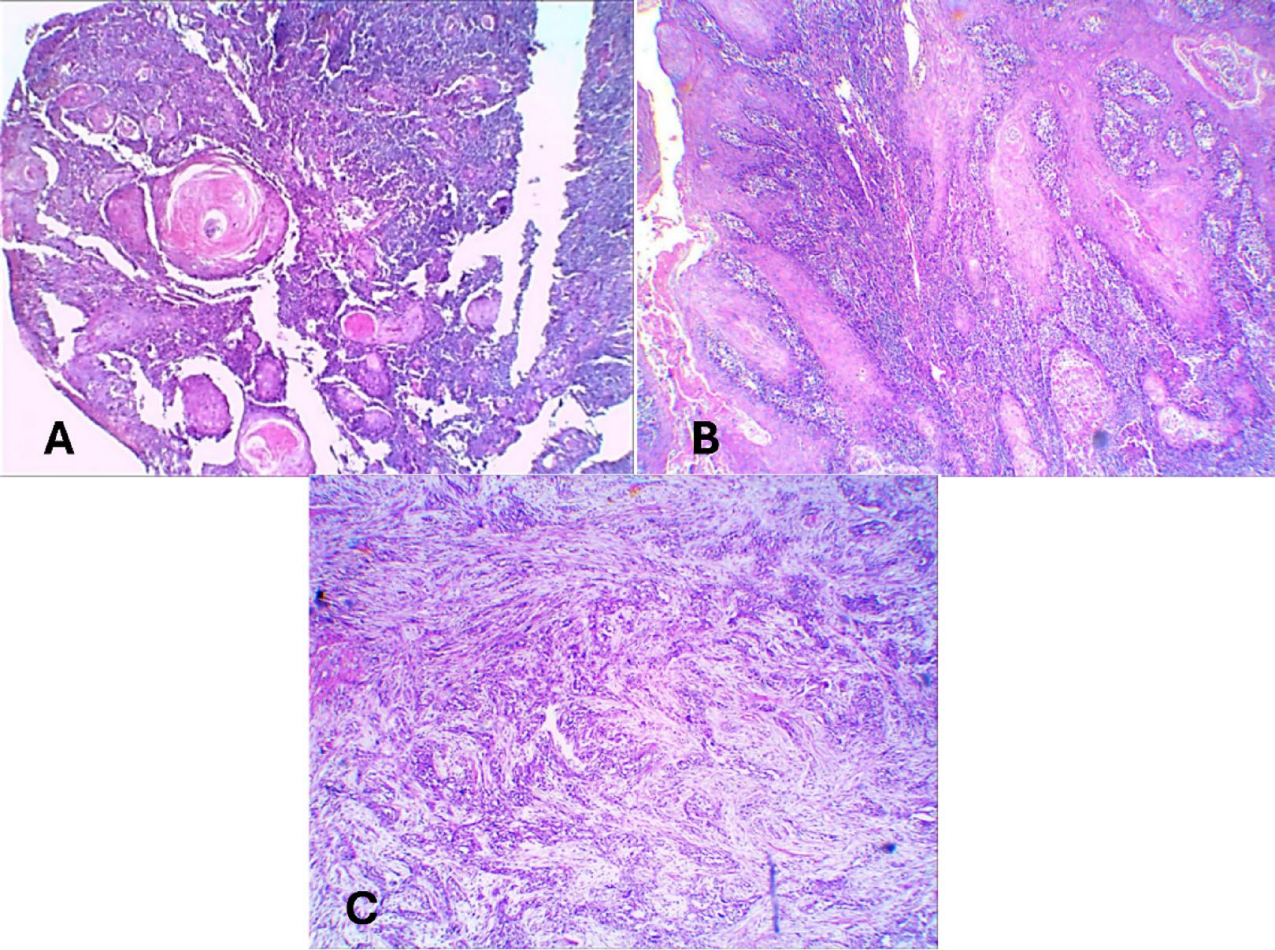
Photomicrographs showing the histopathological grades of the OSCC. (**A**) well-differentiated case showing keratin and epithelial pearls (H&E, x40), (**B**) moderately differentiated case showing epithelial nests (H&E, x40), (**C**) poorly differentiated case showing individually scattered malignant squamous cells (H&E, x40).

**Fig. 3:**
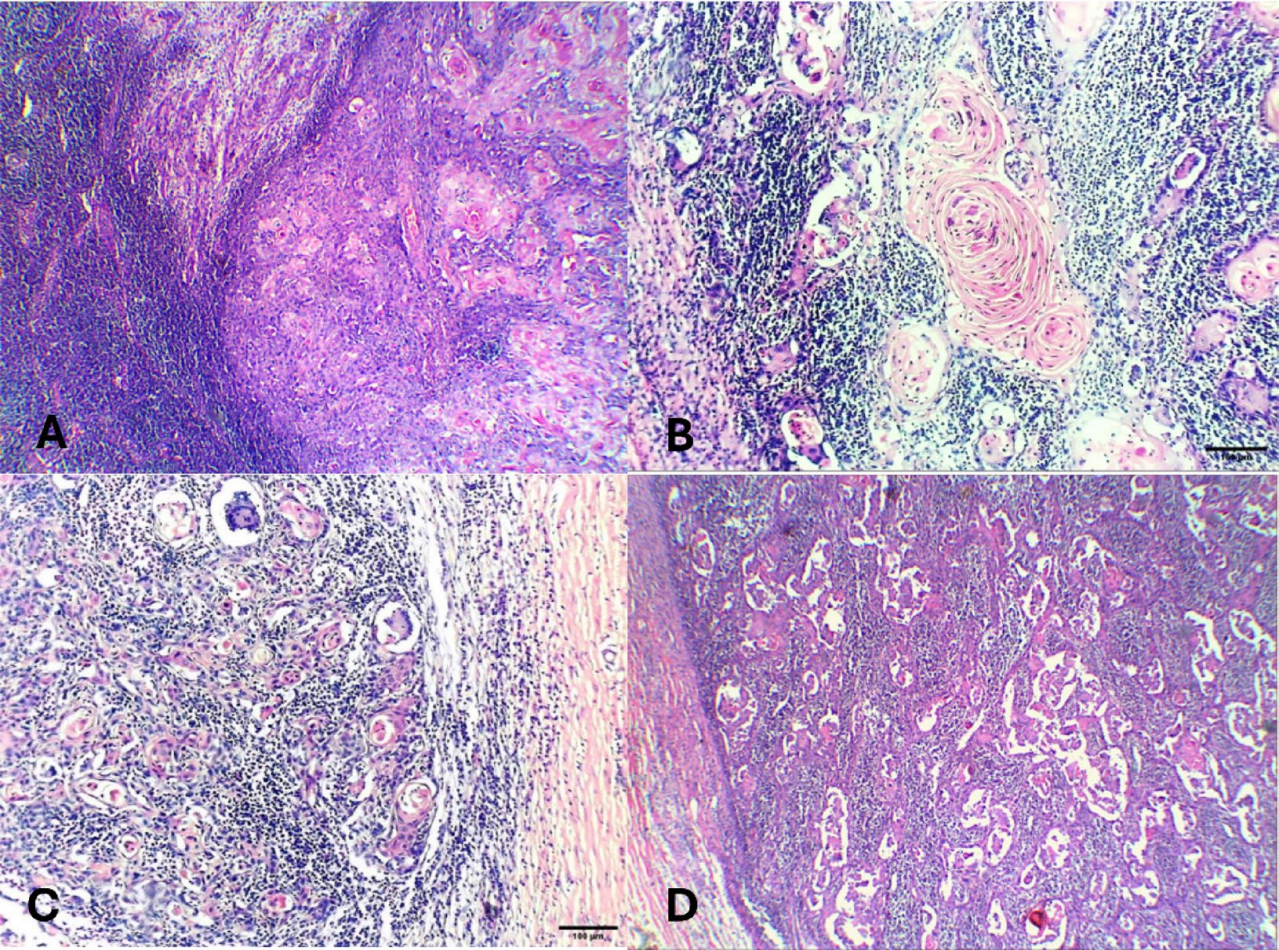
A Photomicrograph showing malignant squamous cells within a lymph node in the form of (**A**) an invading large solid nest (H&E, x40), (**B**) invading moderately sized nests (H&E, x200), (**C**,** D**) infiltrating dispersed small groupings (H&E, x40,100).

As illustrated in Table 2, moderately and poorly differentiated OSCC were the most frequent histological grades (12 cases each), while well-differentiated tumors were less common (6 cases). No statistically significant difference was observed in tumor grading among the studied groups (*p* = 0.199) (Fig. 2). In nodal metastatic cases, tumor cells were observed infiltrating lymph nodes in the form of solid nests, intermediate nests, and dispersed individual cells (Fig. 3).

**Table 2:** Comparison of tumor grade between OSCC studied groups.

Grade	Group A: Primary non-metastatic *n* = 10 (%)	Group B: Primary metastatic *n* = 10 (%)	Group C: Nodal metastatic *n* = 10 (%)	sum	test of significance
Poorly	3(30)	5(50)	4(40)	12	*p* = 0.199
Moderate	3(30)	3(30)	6(60)	12	
Well	4(40)	2(20)	0	6	

Used test: Monte Carlo test, significant where *P* ≤ 0.05.

## Immunohistochemical results

### A- PD-L1 expression in grades of the primary non-metastatic group

**Fig. 4:**
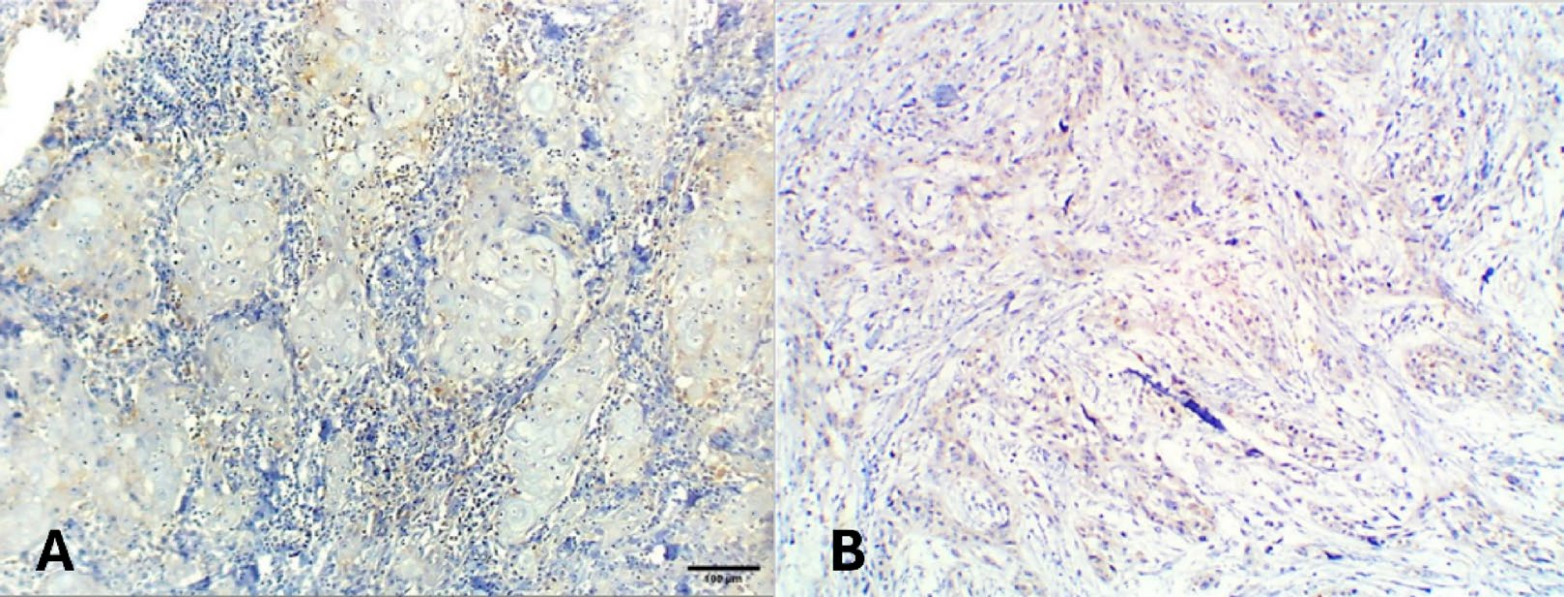
**(A)** A moderately differentiated OSCC showing weak PD-L1 in the epithelial nests (x200), (B) A poorly differentiated OSCC expressed weak PD-L1 in the individually scattered malignant cells (x200).

Positive immunoreactivity was encountered in all (100%) of the non-metastatic OSCC group. Weak PD-L1 expression was observed in all well- and moderately differentiated cases, and most (66.6%) of the poorly differentiated subgroup. PD-L1 expressions were detected in the epithelial cells of nests. No statistically significant association was found between PD-L1 expression and tumor grade (*p* = 0.274) (Table 3; Fig. 4).

**Table 3:** PD-L1 expression among different primary non-metastatic grades.

IH expression code	Grade of Non-metastatic SCC			Test of significance
	well *N* = 4(%)	Moderate *N* = 3(%)	poorly *N* = 3(%)	
Weak strong	4(100) 0	3(100) 0	2(66.7) 1(33.3)	*p = 0.274*

Used test: Monte Carlo test.

### B- PD-L1 expression in grades of primary metastatic cases

**Fig. 5:**
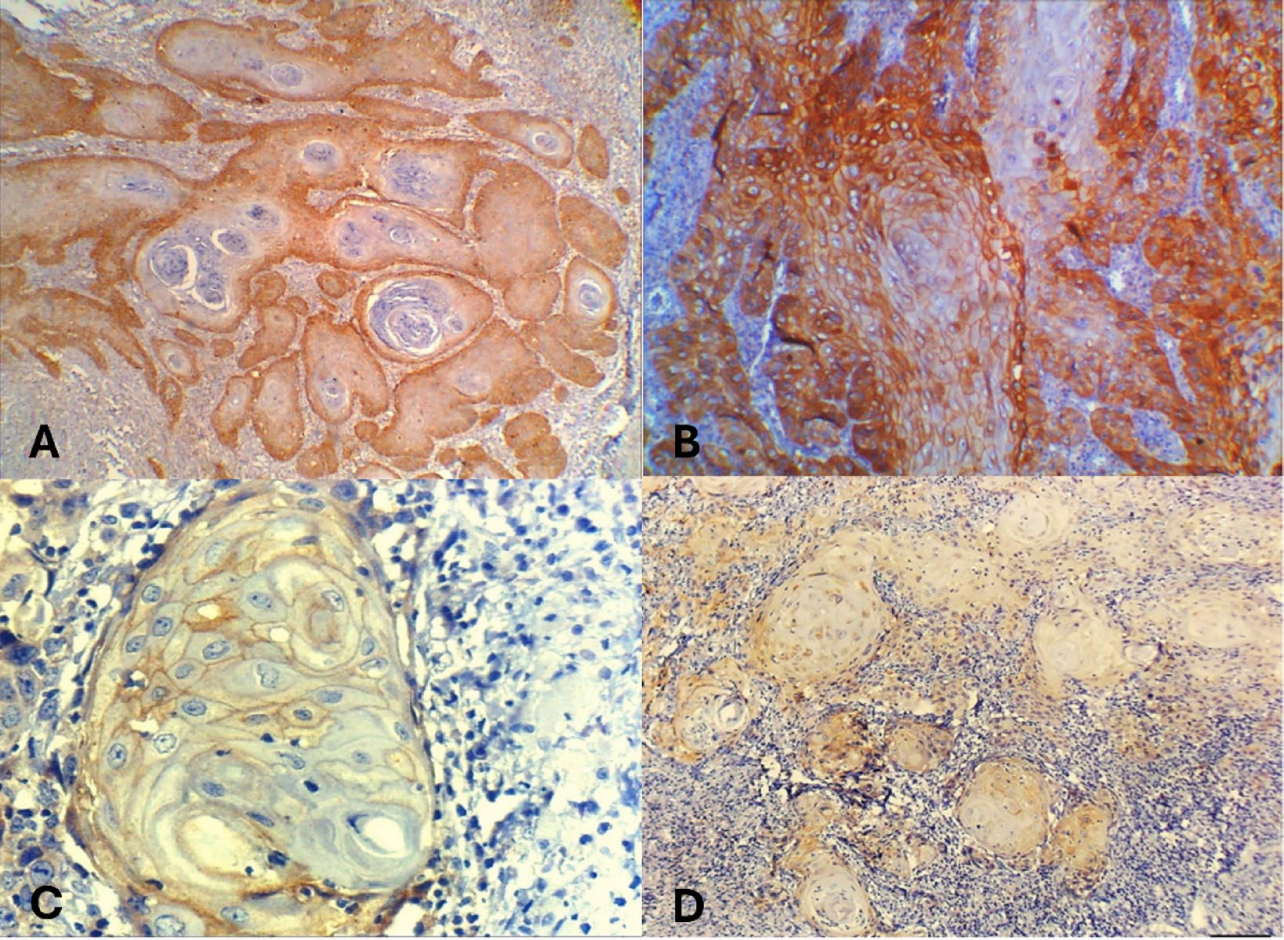
Strong PD-L1 in **(A)**, a case of well-differentiated OSCC. Accentuated at the peripheral cells of the epithelial nest and pearls (x200), (**B**,** C**) a case of well-differentiated OSCC, membranous and cytoplasmic expression (x200, x400), (**D**) epithelial nests of a case of moderately differentiated OSCC (x200).

All cases in this group were positively reactive to the PD-L1 marker. In this group, the staining was mainly strong in all grades: well (100%), moderately (33.3%), and poorly (100%). Two cases of the moderately differentiated grade (66.6%) showed a weak reaction. However, this association was not statistically significant (*p* = 0.054) (Table 4; Fig. 5).

**Table 4:** PD-L1 expression between different grades of primary metastatic OSCC.

IH expression code	Grade of primary metastatic OSCC			Test of significance
	well *N* = 2(%)	Moderate *N* = 3(%)	poorly *N* = 5(%)	
Weak strong	0 2(100)	2(66.7) 1(33.3)	0 5(100)	*p = 0.054*

Used test: Monte Carlo test.

### C- PD-L1 expression findings in grades of nodal metastatic cases

**Fig. 6:**
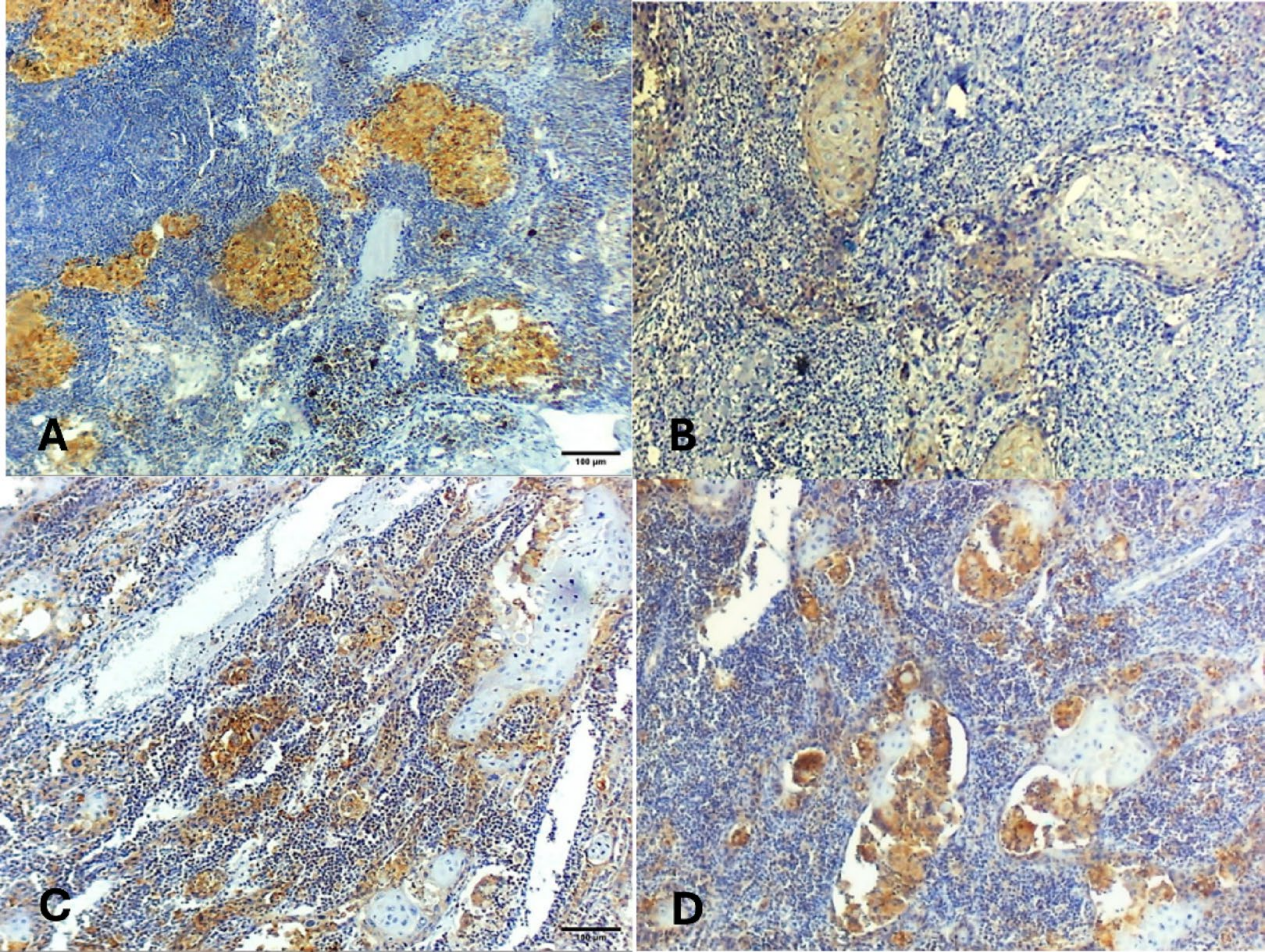
**(A**,** B**,** C)** Strong PD-L1 in OSCC metastasizing in lymph node (x100, x200, x200), (**D**) Strong PD-L1 reaction of malignant cells with lymphovascular infiltration.

All cases involved in this group were positively reactive to the PD-L1 marker. Strong PD-L1 expression was observed in 83.3% of the moderately differentiated and all well-differentiated cases. PD-L1 expression was high in the epithelial squamous nests that are dispersed in lymph nodes. However, this association was not statistically significant (*p* = 0.400) (Table 5; Fig. 6).

**Table 5:** PD-L1 expression between different grades of nodal metastatic cases.

IH expression code	Grade of nodal metastatic OSCC		Test of significance
	Poorly *N* = 4(%)	Moderate *N* = 6(%)	
Weak strong	0 4(100)	1(16.6) 5(83.3)	*p = 0.400*

Used test: Fisher exact test.

### D- PD-L1 expression in relation to clinical parameters

Among the clinical parameters investigated (age, gender, site, and pain), PD-L1 expression showed no statistically significant association, except for age, whereas higher PD-L1 expression was represented in older patients (*p* = 0.024) (Table 6).

**Table 6:** PD-L1 expression in relation to clinical parameters.

	IH expression code			Test of significance
	absent	weak	strong	
Age/years	50 ± 8.66	48.47 ± 12.77	60.22 ± 12.94	*P* = 0.024*
Sex				
Male	1 (33.3)	10(52.6)	6(33.3)	*P* = 0.468
Female	2 (66.7)	9(47.4)	12(66.7)	
Sites				*p* = 0.605
Cheek	1(33.3)	5(26.3)	6(33.3)	
Mandible	1(33.3)	2(10.5)	2(11.1)	
Palate	1(33.3)	2(10.5)	4(22.3)	
Tongue	0	10(52.6)	6(33.3)	
pain	1(33.3)	10(52.6)	7(38.9)	*p* = 0.643

Used test: Monte Carlo test, significant where *P* ≤ 0.05.

### E- Final scoring of PD-L1 IH expression

PD-L1 expression in normal control tissues was either absent or showed minimal weak immunoreactivity, in contrast to the increased expression observed in OSCC cases. While all cases of OSCC were immunoreactive for PD-L1, the strong final PD-L1 immunoreactivity score was most observed in the nodal metastatic group (90%). The proportion of strongly positive cases decreased progressively among the other studied groups from the primary metastatic OSCC (80%) to the non-metastatic OSCC (10%). Inter-observer agreement between the two evaluators showed substantial concordance (κ = 0.71), indicating reliable assessment of PD-L1 immunostaining. A statistically significant difference in final PD-L1 immunoreactivity scores was observed among the studied groups (*p* < 0.001) (Table 7).

**Table 7:** Comparison of final PD-L1 IH score of expression in the studied groups.

	Non-metastatic *n* = 10(%)	Metastatic OSCC *n* = 10(%)	LN metastatic OSCC *n* = 10(%)	test of significance
-Ve	0	0	0	*p* < 0.001* κ = 0.71
Weak	9(90)	2(20)	1(10)	
Strong	1(10)	8(80)	9(90)	

Used test: Monte Carlo test, significant where *P* ≤ 0.05.

## Discussion

OSCC is a malignant disease caused by multiple factors originating in the oral mucosa and has a poor prognosis that has changed minimally in the last few decades. In OSCC, lymph node metastasis (LNM) is a significant indicator of cancer-related outcomes^5^. Regarding the age in the current investigation, the majority of cases were presented between 38 and 67 years, with a mean of 58.50 years, and this was within the range reported by other studies^27–29^. The high incidence in a much younger age group reported by other investigators, as Mneimneh^30^ might be due to the increased exposure to risk factors among this age group of age, relevant to variable social cultures.

Concerning gender, a slight female tendency was encountered. This observation was consistent with^31^ and Elmetwaly et al.^32^ in spite of being contradictory to the generally accepted higher incidence^27,33,34^ in males than in females. This discrepancy could be explained by regional differences in exposure to risk factors, variations in lifestyle habits, and the limited sample size of the current study, which may influence the observed gender distribution.

As regards the site, the most presently affected anatomical site was the tongue. Afterward, the buccal mucosa as well as the palate. This was in line with other investigators’ reports of OSCC in Egypt^35^ and in other developing countries^24,36^. Contradictory results were documented in Taiwan, wherein the buccal mucosa and the tongue were the most common sites, respectively^37^. Whereas in Brazil, the most common location was the lip^38^. There’s a worldwide variability in findings documented in various studies concerning the most affected site of OSCC in the oral cavity. These variations may be explained by geographic differences in risk factor exposure, including tobacco habits, alcohol consumption, betel quid chewing, and ultraviolet radiation, which influence the most affected anatomical site of OSCC among different populations^39^.

Regarding pain, almost half of the cases were painless, and the other half were painful. Khawaja revealed in his study that almost all of the cases were painful^40^. But Albano revealed that some OSCC cases present without pain in early stages, while pain increases with lesion size and advanced disease^41^. this discrepancy may be attributed to the small sample size engaged in the current study.

About OSCC histological grades, most of the presented cases were of the moderate and well-differentiated grades, whereas the poorly differentiated type was the least common type. This was in accordance with studies^35,42,43^, which found that the majority of cases were well and moderate differentiated OSCCs. However, some researchers have reported variable results^44^, who reported that the poorly differentiated was more common than the well differentiated grade. This might be explained by the variable environmental carcinogenic factors, such as tobacco, alcohol, and viral infections, and the role of genetic factors has been suggested.

OSCC has been considered to be a very immunosuppressive cancer, and there is increasing recognition that the dysfunction of the immune system might be a contributing factor to treatment failure of OSCC cases. Inhibitory signaling pathways known as immunological checkpoints mediate immune tolerance. The axis PD-1/PD-L1 is one of the very important immunologic axes^19^. PD-L1 plays a central role in tumor immune evasion in OSCC through modulation of the PD-1/PD-L1 immune checkpoint pathway. PD-L1 is expressed on tumor cells and tumor-infiltrating immune cells and interacts with the PD-1 receptor on activated cytotoxic T lymphocytes. This interaction transmits inhibitory signals that lead to T-cell exhaustion, inhibition of cytotoxic activity, and escape from immune surveillance. As a result, tumor cells escape immune surveillance and continue to proliferate, thereby facilitating tumor progression and metastasis^45^. In OSCC, PD-L1 expression is often upregulated in response to inflammatory cytokines within the tumor microenvironment, particularly interferon-gamma (IFN-γ), a phenomenon known as “adaptive immune resistance.” This reflects a feedback mechanism in which active anti-tumor immune responses paradoxically induce immune checkpoint activation, allowing tumor cells to survive under immune pressure^46^.

Moreover, increased PD-L1 expression has been associated with epithelial–mesenchymal transition (EMT), enhanced tumor invasiveness, and promotion of metastatic potential through modulation of immune escape pathways and interaction with oncogenic signaling pathways^47^. PD-L1 is a transmembrane protein that is thought to be a co-inhibitor of the immune response. Additionally, it plays a critical role in several malignancies by attenuating the host immune reaction to tumor cells^20^.

During the investigation of the current studied cases, there was a notable increase in PD-L1 in the lymph node metastatic group compared with the primary metastatic and non-metastatic groups, respectively. This pattern suggests that PD-L1 upregulation may be associated with tumor progression and metastatic potential rather than normal tissue expression. This finding is biologically plausible, as increased PD-L1 expression may reflect an adaptive immune resistance mechanism in response to anti-tumor immune pressure within the tumor microenvironment^48^. Furthermore, the higher PD-L1 expression observed in metastatic lesions supports its potential role in facilitating immune escape during lymph node dissemination^49^. Also, this may suggest that PD-L1 expression is more closely related to tumor immune microenvironment and metastatic behavior rather than histological differentiation alone. Strong immunoreactivity of PD-L1 among the malignant cells and nests was evident in most cases of primary SCC with metastasis and L.N metastatic SCC groups, whereas the majority of the non-metastatic SCC group showed weak immunoreactivity. This result follows previous studies by^26,50,51^. Regional lymph nodes are crucial because they serve as anatomic barriers that prevent tumor cells from systemic dissemination, and this represents the host immune response. Accordingly, these findings imply that PD-L1 expression may be associated with lymph node metastasis and poor prognosis in patients with OSCC. The clinical significance of PD-L1 expression has gained increasing attention in recent years because of its role in immunotherapy targeting the PD-1/PD-L1 axis^52^. Immune checkpoint inhibitors such as Pembrolizumab and Nivolumab have demonstrated encouraging therapeutic outcomes in patients with recurrent or metastatic OSCC^53^. These agents function by blocking the inhibitory interaction between PD-1 receptors on T lymphocytes and PD-L1 expressed on tumor cells, thereby restoring anti-tumor immune responses. Consequently, assessment of PD-L1 expression has been widely investigated as a potential biomarker to help guide immunotherapeutic strategies in head and neck cancers^15,54^. Although the present study did not include survival or treatment-response data, the observed pattern of PD-L1 expression in OSCC highlights the biological relevance of this immune checkpoint pathway and supports the growing interest in incorporating immune-based therapeutic approaches in the management of OSCC. Therefore, comprehensive detection and evaluation of the prognostic factors are crucial for improved management and therapy of OSCC patients^15,54^.

Our results contradict those of others, who failed to disclose any significant correlation between PD-L1 expression and metastatic disease^55,56^. Such discrepancies may be explained by differences in antibody clones, scoring systems, cut-off definitions, tumor heterogeneity, and sample size across studies. In our study, there was no statistically significant difference between the expression of PD-L1 and different grades of OSCC in each group. This might be attributed to the small sample employed in the present study in each group. These findings were in concordance with a study performed by Greeshma et al.^34^. Regarding the relation between clinicopathological parameters and the PD-L1 of the cases studied, there was only a statistically significant correlation in the high-age group. This finding may reflect age-related immune modulation and immunosenescence, which could enhance tumor immune evasion. Meanwhile, the PD-L1 expression failed to reveal any statistically significant difference between the other clinical parameters. This was in accordance with the study of de Vicente et al.^51^.

The significant difference observed when evaluating PD-L1 using the combined scoring system (percentage and intensity) may indicate that this method provides a more comprehensive assessment of PD-L1 expression in OSCC. The same significant association was obtained upon evaluating the PD-L1 expression via estimating the intensity as well as the percentage of the positively reactive cells in the current study groups. This supports the robustness of combined scoring in capturing biologically relevant differences in immune checkpoint expression. This was in keeping with the reports of^34,57,58^ but in disagreement with Kamiya et al.^59^, who concluded that it was the intensity of PD-L1 alone, but not the percentage of PD-L1-positive cells, that was significantly different among the OSCC grades.

### Limitations

This study has several limitations that should be acknowledged. The relatively small sample size (*n* = 30), with equal distribution across three groups, may limit the statistical power of the findings. In addition, the retrospective study design may introduce selection bias due to reliance on archived data.

Furthermore, the absence of survival or longitudinal follow-up data restricts the ability to draw definitive conclusions regarding the prognostic value of PD-L1 expression. Lastly, as this is a single-center study, the generalizability of the findings to broader populations may be limited. Therefore, further large-scale, multicenter studies with longitudinal follow-up are recommended to validate these results.

## Conclusions

PD-L1 expression was higher in metastatic OSCC, particularly in lymph node involvement, and correlated with depth of invasion, suggesting a role in tumor progression. Its expression showed a significant association with patient age but not with other clinicopathological parameters or tumor grade. Combined scoring methods appeared more reliable for PD-L1 evaluation. Larger studies are needed to confirm these findings.

## Data Availability

The datasets used and/or analyzed during the current study are available from the corresponding author on reasonable request.

## Abbreviations

DAB: Diaminobenzidine
FFPE: Formalin-fixed paraffin-embedded
IHC: Immunohistochemistry
IL10: Interleukin-10
OSCC: Oral squamous cell carcinoma
LNM: Lymph node metastasis
PD-1: Programmed cell death 1
PD-L1: Programmed cell death ligand 1
PBS: Phosphate-buffered saline
TIL: Tumor-infiltrating lymphocytes
TNM: Tumor -node- metastasis
WHO: World health organization
